# The future is now: Using the lessons learned from the ACTIV COVID-19 therapeutics trials to create an inclusive and efficient clinical trials enterprise

**DOI:** 10.1017/cts.2024.622

**Published:** 2024-10-15

**Authors:** Stacey J. Adam, Sarah E. Dunsmore, Lisa H. Merck, Sarah W. Read, Yves Rosenberg

**Affiliations:** 1 Foundation for the National Institutes of Health, North Bethesda, USA; 2 National Center for Advancing Translational Sciences, National Institutes of Health, Bethesda, USA; 3 Department of Emergency Medicine, Virginia Commonwealth University, Richmond, Virginia, USA; 4 National Institute of Allergy and Infectious Diseases, National Institutes of Health, Bethesda, USA; 5 National Heart, Lung and Blood Institute, National Institutes of Health, Bethesda, USA

## Introduction

The COVID-19 public health emergency (PHE) has been unprecedented in its impact on global health. Likewise, the research response to the PHE has been unparalleled, with government agencies, academic institutions, biomedical and pharmaceutical companies, and philanthropic organizations pivoting from other research pursuits to collectively study SARS-CoV-2, the COVID-19 causative agent, and develop diagnostics, therapeutics, and vaccines for detection, treatment, and disease prevention.

By March 11, 2020, SARS-CoV-2 infections were so pervasive that the World Health Organization declared a state of global pandemic. At the time of declaration, US healthcare providers were struggling to implement SARS-CoV-2 countermeasures, and there were no known effective therapies. This lack of therapeutics drove the launch of hundreds of well-intentioned but poorly coordinated and underpowered studies that failed to yield clinically meaningful results [1]. To address the lack of coordination and accelerate therapeutic evaluations for COVID-19, the National Institutes of Health (NIH) and its collaborators launched a public-private partnership (PPP) called Accelerating COVID-19 Therapeutic Interventions and Vaccines (ACTIV) in April 2020 [2]. One goal of the PPP was to design and implement platform trials for rapid evaluation of candidate therapeutics against COVID-19. Ultimately, 11 master protocols were launched to assess 37 agents of different classes spanning diverse patient settings. The trials were designed to structurally adapt over time to assimilate increased knowledge of viral pathogenesis and incorporate the evolution of disease manifestations resulting from the emergence of novel viral variants and implementation of vaccines and treatment modalities.

**Figure 1.**
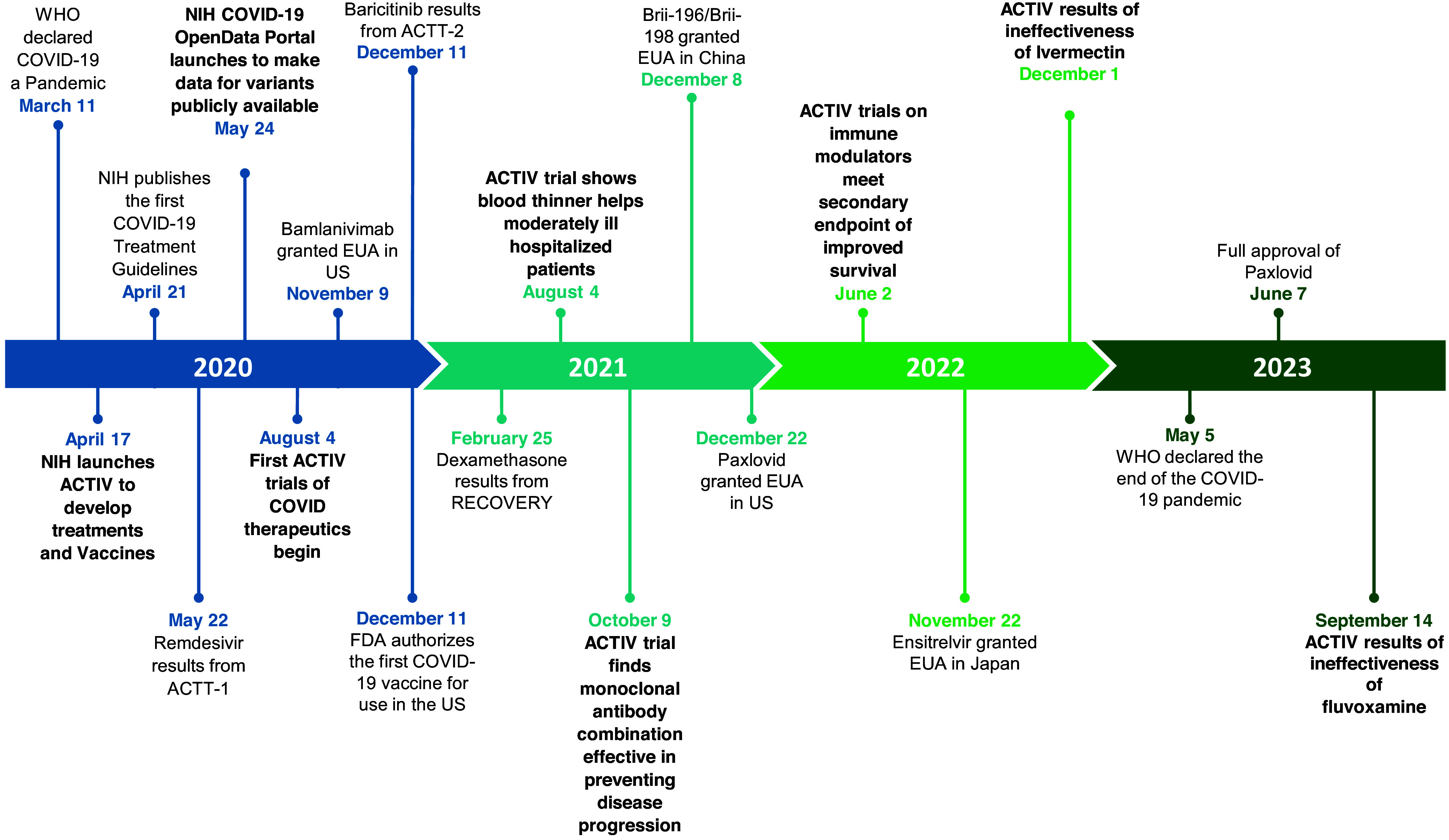
Timeline of COVID-19 therapeutic milestones.

As vaccine availability increased and later viral variants induced less severe disease, hospitalizations sharply decreased, and ACTIV inpatient trials began to conclude in early 2022. The PHE ended on May 11, 2023, yet ACTIV outpatient trials continued to test new agents for symptom management into 2024. A timeline summary of the relevant therapeutics across the pandemic is presented in Figure 1.

## Description of themed issue

**Figure 2.**
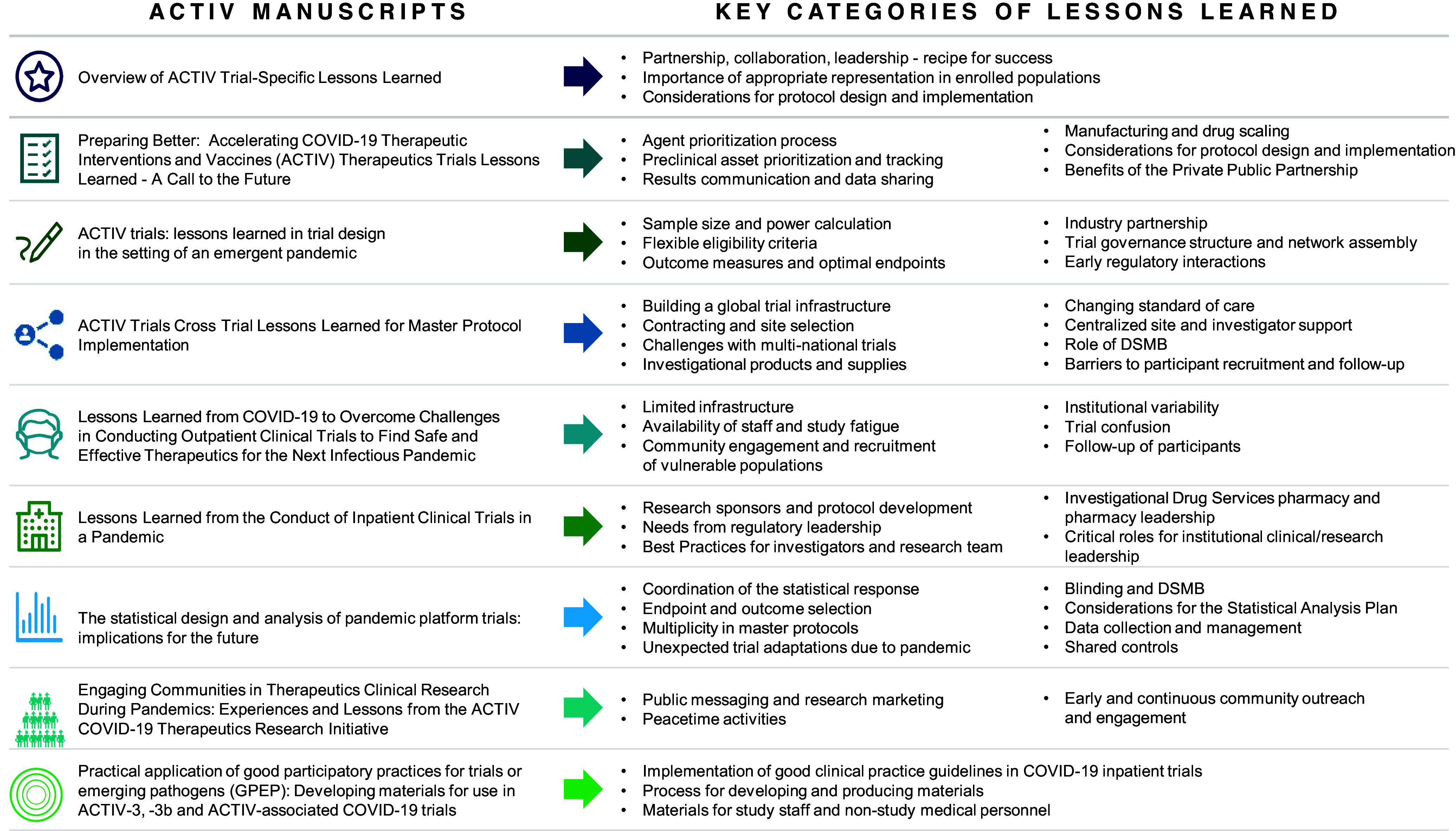
Key lessons learned themes discussed in each of the ACTIV manuscripts. ACTIV = Accelerating COVID-19 Therapeutic Interventions and Vaccines; DCR = Department of Clinical Research; DSMB = Data and Safety Monitoring Board; GGP-EP = good participatory practice guidelines.

In this supplemental issue, the ACTIV Therapeutics-Clinical Working Groups (WGs) and Clinical Trial Teams document valuable lessons believed to benefit future healthcare leaders and researchers faced with preparing for and responding to global PHEs. Here, we present brief summaries from manuscripts that describe aspects of the design and implementation of master protocols; inpatient/outpatient site-specific challenges; statistical considerations for innovative, adaptive master protocols; and important approaches to community engagement. These findings inform lessons that pinpoint the importance of incentivizing collaboration through centralized resources, community outreach for efficient and equitable enrollment of populations most affected by PHEs, and global clinical trial collaboration. Implementation of these lessons, listed in Figure 2, will enhance global preparedness for the next PHE.

Acting as primers for readers to fully understand lessons shared in other articles, Draghia-Akli *et al.* describe ACTIV PPP efforts to identify effective treatments for SARS-CoV-2 and COVID-19 symptoms. Providing an executive view on ACTIV’s therapeutic efforts, Adam *et al.* describe the WGs’ efforts to assess over 800 potential therapeutic agents [3], selecting 37 drug formulations for deployment across 11 platform trials designed by the WGs and trial teams [4]. With 620 sites globally, over 26,000 patients were enrolled in these 11 master protocols.

Elaborating on ACTIV trial master protocol design, Jahromi *et al.* detail cross-protocol themes, including the sample size and power calculation, need for flexible eligibility criteria, selection of outcome measures and optimal endpoints, value of industry partnership, trial governance and network assembly, and criticality of early regulatory interactions. In a separate manuscript, Jahromi *et al.* present lessons for master protocol implementation, highlighting the importance of international regulatory body cross-communication; diverse patient engagement; universal outcomes platforms assembly; central data and biospecimen repositories; central resources for startup, training, and study monitoring; and the designation of resources for drug manufacturing/delivery to support interventions. Additionally, the team calls for the establishment of a US clinical research agenda that includes the maintenance of readily deployable clinical trial infrastructure.

Site-specific challenges in trial performance in inpatient and outpatient settings are presented by O’Halloran *et al. and* Oh *et al., respectively.* Universally, initial trial execution was challenged by infection control measures and limited availability of personal protective equipment (PPE). In response, many sites implemented electronic patient education and consent procedures, as well as remote patient monitoring. Both trial types dealt with clinically stretched resources and provider fatigue. However, unique to the outpatient cohort, limited infrastructure and trained personnel shortages impacted patient recruitment, enrollment, and follow-up. Inpatient sites adapted quickly to the evolving interface between clinical care and research: medication administration and laboratory draws timed to available clinical resources, and tools were developed to stratify enrollments across coinciding clinical trials.

Statistical considerations in platform trial development are outlined by Lindsell *et al*. for the ACTIV Cross-Trial Statistics Group. The PPP facilitated rapid response, protocol development, and adaptive design components. Across protocols, strict interim analyses and Bayesian algorithms informed early discontinuation of ineffective therapeutics in favor of new agents. Flexibility and trial adaptations were implemented to address temporal changes in disease severity, prevalence, population immunity, and evolving standards of care.

The importance of patient/community engagement is emphasized by Wohl *et al.* Authors highlight limitations to engagement including infection control requirements, community perceptions, political divisions, and variable penetrance of media/social media efforts across age, race, ethnic, and geographic demographics. The authors endorse activities such as comprehensive public messaging, interfacing with patients at hospitals and outpatient centers, and collaboration with teams harboring pre-established community relationships. While Wohl *et al.* focus mainly on outpatient recruitment, Marines-Price *et al.* provide an exemplar with the ACTIV-3 trial on Good Participatory Practice for inpatient studies during a PHE and peacetime. They detail standard and novel tools for study conduct, patient recruitment, and informed consent among others.

## Time capsule: messages to the future

In this special issue, the ACTIV PPP highlighted the strengths of coordinated research strategies, flexible master protocol frameworks, and collaborative governance structures involving all relevant stakeholders. Further, ACTIV trial teams demonstrated exemplary clinical trial performance under extreme PHE conditions, which stretched the healthcare ecosystem to and beyond its breaking point. The ACTIV PPP garnered many successes; however, it also revealed, and failed to fully address, shortcomings in the healthcare ecosystem that must be rectified prior to the next PHE for an improved global response.

As noted in this issue, pandemic response efforts revealed the US clinical trial system places too much focus on large healthcare centers, retains uncoordinated efforts across sites and networks, lacks reach and ability to scale quickly into underserved population communities, and poorly incentivizes investigator collaboration. These shortcomings prevent the system from pivoting quickly during PHEs and cause trials to run inefficiently in peacetime. To improve future PHE response trajectories, we highlight a few critical challenges remaining in the clinical trial ecosystem below.

### Incentivize collaboration through centralization of key resources

The healthcare system was unprepared to face supply chain shortages early during this PHE. Frontline providers faced shortages of PPE, COVID-19 testing equipment, ventilator supplies, and medications. To be better prepared for the next PHE, domestic manufacturing capabilities must be established to enable supply chain control, allowing for fabrication efforts to be able to pivot when equipment demand fluctuates for both clinical trials and standard care.

Further highlighting the necessity of centralized collaboration, as of February 9, 2024, the United States Center for Disease Control estimates 1,176,639 deaths in the USA due to COVID-19. Unfortunately, this number is a gross underrepresentation due to poor patient tracking. For example, while mortality in Florida reached the highest reported in years, health officials lacked access to COVID-19 testing early, preventing the quantification of true disease prevalence [5].

### Sustain local community engagement

ACTIV trials demonstrated efficient enrollment because they were deployed within existing NIH trial networks with extensive infrastructure, often focusing on large institutions versus community care centers. However, a challenge to clinical trials is the recruitment of diverse participants who are traditionally underrepresented, a well-documented and long-standing concern [7]. The social inequities and structural racism driving underrepresentation in clinical trials were glaringly apparent throughout the COVID-19 pandemic, often fueled by mistrust, political divisions, and community unawareness. Therefore, it is essential to conduct community-led health equity research, support structural interventions, and form partnerships to combat institutional racism that sustains barriers to clinical trial participation [8]. Community members should be engaged in all stages of research design, not just invited to post-design completion or recruitment focus groups. The Wohl *et al.* paper emphasizes early public engagement to earn trust as a critical component of successful therapeutic trial implementation, as has been successful in other countries [9].

To further diversify representation within clinical trials, inclusive networks extending beyond tertiary and quaternary care centers, community health centers, and primary care settings must be built [10]. The Director of the NIH, Monica Bertagnolli, agrees the NIH faces challenges with conducting research in settings where Americans receive treatment and care [11]. By forging trial networks that seek to include individuals from diverse backgrounds, public health equity can be enhanced.

### Global Clinical Trial Collaboration

As noted in this issue, ACTIV trials were components of a larger global response to the COVID-19 pandemic and benefited from information exchange with other platform trials such as REMAP-CAP [12], I-SPY-COVID [13], RECOVERY [14], Solidarity [15], and others. However, future pandemic responses could be bolstered by furthering collaboration and implementation of common global regulatory frameworks for all aspects of therapeutics development, including harmonized data collection, centralized biospecimen and data storage, and shared site management strategies. The Strategies and Treatments for Respiratory Infections and Viral Emergencies (STRIVE) network is an example of this trial infrastructure established to support future research responses during a pandemic [16], as noted in Jahormi *et al*.

Overall, as the clinical trial ecosystem relies on components listed above like domestic manufacturing, centralized resources, community inclusion, diversification of clinical trial networks, and global collaboration, our ability to address these challenges now will influence the rigor of future PHE responses. The pursuit of improving these resources now will enable innovations to streamline clinical trial practice both during PHEs and standard care.
